# The urinary-metabolite-based lung cancer index (uLCI): an interpretable machine-learning risk model for early-stage disease

**DOI:** 10.64898/2026.06.26.26356700

**Published:** 2026-06-29

**Authors:** Mohammed A Khan, Ewy A Mathé, Sharon R Pine, Frank J Gonzalez, Curtis C Harris, Xin W Wang, Daxesh P Patel

## Abstract

**Background:**

Five-year survival from lung cancer exceeds 60% at stage I–II but falls below 10% once metastasis occurs. Low-dose CT (LDCT) screening reduces mortality in heavy smokers but carries a false-positive rate of approximately 29% and is restricted to smoking-based eligibility, leaving most cases undetected. We aimed to develop and independently validate an interpretable machine-learning urinary metabolite risk index (uLCI) for non-invasive lung cancer detection.

**Methods:**

Four urinary metabolites—creatine riboside (CR), N-acetylneuraminic acid (NANA), 27-nor-5β-cholestane-3α,7α,12α,24,25-pentol (CP), and cortisol sulfate (CS)—and three clinical variables (age, race, smoking) were integrated by Lasso-regularised logistic regression into a uLCI score. The model was developed under 10-fold cross-validation in the NCI-Maryland (NCI-MD) cohort (n=845; 470 controls, 375 cases, stages I–IV) and applied without refitting to the independent Colorado Lung Cancer Cohort (n=488; 211 controls, 277 cases). Analyses were prespecified; reporting followed TRIPOD+AI.

**Findings:**

uLCI achieved an area under the curve (AUC) of 0·906 (95% CI 0·887–0·926) in NCI-MD and 0·748 (0·701–0·793) in the independent Colorado cohort. Scores rose monotonically across stages in both cohorts (Spearman ρ=0·69 and 0·45; both p<0·0001). Stage-specific discrimination was preserved from stage I to IV (NCI-MD 0·900–0·927; Colorado 0·722–0·843). Net reclassification improvement over clinical variables was 1·24 (1·14–1·36) and 0·74 (0·56–0·90). uLCI tertiles stratified post-resection survival in stage I–II disease (adjusted hazard ratio 2·03, 1·26–3·27).

**Interpretation:**

uLCI is an independently validated, interpretable urinary risk index that detects lung cancer across all stages, with monotonic stage progression and post-resection prognostic value. Its false-positive rate compares favourably with published estimates for LDCT and cell-free-DNA assays, supporting prospective head-to-head evaluation as a non-invasive triage tool, including in screening-ineligible populations.

**Funding:**

Intramural Research Program, Center for Cancer Research, National Cancer Institute, US National Institutes of Health.

**Research in context:**

**Evidence before this study**

We searched PubMed, Embase, and Web of Science from Jan 1, 2000, to Jan 31, 2026, without language restriction, for biomarker-based diagnostic or risk models for lung cancer, using “lung cancer”, “early detection”, “biomarker”, “urine”, “metabolite”, “machine learning”, “TRIPOD”, and “validation in an independent cohort”. Five-year survival exceeds 60% at stage I–II but falls below 10% after metastasis. Low-dose CT reduces mortality in heavy smokers but carries an approximately 29% false-positive rate and excludes never-smokers, who account for 10–25% of US lung cancers and up to 40% globally; blood-based cell-free DNA and methylation assays report rates near 27%. Most prior urinary-metabolite work, including our 2024 creatine-riboside and N-acetylneuraminic-acid report, used case–control designs without a locked, integrated model, and—even where two cohorts were analyzed—lacked prespecified independent validation or TRIPOD+AI-compliant reporting; an interpretable urinary index integrating an expanded metabolite panel with clinical variables under these standards had not been described.

**Added value of this study**

Across two cohorts (1333 individuals), we developed and independently validated uLCI, an interpretable Lasso-regularised logistic index combining four urinary metabolites (CR, NANA, CP, CS) with age, race, and smoking, reported to TRIPOD+AI standards. The prespecified locked model achieved an AUC of 0·906 (95% CI 0·887–0·926) in NCI-MD development and 0·748 (0·701–0·793) in the independent Colorado cohort without refitting, with discrimination preserved from stage I to IV (0·900–0·927; 0·722–0·843). uLCI rose monotonically with stage in both cohorts (Spearman ρ=0·693 and 0·449; both p<0·0001), improved reclassification over clinical variables (net reclassification improvement 1·24 and 0·74), and independently stratified post-resection survival in stage I–II disease (adjusted hazard ratio 2·03 in NCI-MD; 3·81 in Colorado). On indirect benchmarking, the 17–19% false-positive rate was below published estimates for low-dose CT (∼29%) and DELFI (∼27%), and discrimination was preserved in never-smokers — whom low-dose CT excludes — and across racial subgroups in the diverse development cohort.

**Implications of all the available evidence**

A locked, independently validated, non-invasive urinary index that is interpretable, accurate from stage I, stage-responsive, and prognostic after resection addresses a defined detection gap, with a plausible role as a low-cost triage test that raises pre-test probability before imaging and extends risk assessment to screening-ineligible never-smokers, complementary to low-dose CT. Prospective screening-cohort evaluation (planned in the NCI PLCO and Southern Community Cohort biobanks), head-to-head comparison with blood-based assays, recalibration to screening prevalence, and replication in diverse cohorts are the warranted next steps.

## Full Text Availability

The license terms selected by the author(s) for this preprint version do not permit archiving in PMC. The full text is available from the preprint server.

